# Signal transducer and activator of transcription 3 activation up-regulates interleukin-6 autocrine production: a biochemical and genetic study of established cancer cell lines and clinical isolated human cancer cells

**DOI:** 10.1186/1476-4598-9-309

**Published:** 2010-12-02

**Authors:** Wei-Lun Huang, Hsuan-Heng Yeh, Chien-Chung Lin, Wu-Wei Lai, Jang-Yang Chang, Wen-Tsan Chang, Wu-Chou Su

**Affiliations:** 1 Institute of Basic Medical Sciences, College of Medicine, National Cheng Kung University, Tainan 704, Taiwan; 2 Institute of Clinical Medicine, College of Medicine, National Cheng Kung University, Tainan 704, Taiwan; 3 Department of Biochemistry and Molecular Biology, College of Medicine, National Cheng Kung University, Tainan 704, Taiwan; 4 Department of Internal Medicine, National Cheng Kung University College of Medicine and Hospital, Tainan 704, Taiwan; 5 Department of Surgery, National Cheng Kung University College of Medicine and Hospital, Tainan 704, Taiwan; 6 National Institute of Cancer Research, National Health Research Institutes, Tainan 704, Taiwan; 7 Center for Gene Regulation and Signal Transduction Research, National Cheng Kung University, Tainan 704, Taiwan

## Abstract

**Background:**

Spontaneous interleukin-6 (IL-6) production has been observed in various tumors and implicated in the pathogenesis, progression and drug resistance in cancer. However, the regulation of IL-6 autocrine production in cancer cells is not fully understood. IL-6 is auto-regulated in many types of cell. Two of the three major downstream pathways of IL-6, MEK/extracellular signal-related kinase (Erk) pathway and phosphatidylinositol 3-kinase (PI3-K)/Akt pathway, have been shown to regulate IL-6 expression through the activation of AP-1 and NF-κB. However, it is not clear what the role of Janus kinase (Jak) 2/signal transducer and activator of transcription (Stat) 3 pathway. This study was designed to determine the role of Jak2/Stat3 pathway in the regulation of IL-6 autocrine production in cancer cells.

**Results:**

Inhibitors of Jak2/Stat3, MEK/Erk and PI3-K/Akt pathways down-regulated IL-6 secretion in the lung adenocarcinoma PC14PE6/AS2 (AS2) cells, which spontaneously secreted IL-6 and possessed constitutively activated Stat3. Transfection with dominant-negative Stat3, Stat3 siRNA, or Stat3 shRNA decreased IL-6 expression in AS2 cells. Conversely, transfection with constitutively-activated Stat3 increased the production of IL-6. In AS2 derived cells, resistance to paclitaxel was positively correlated with Stat3 activation status and the expression of IL-6, which is commonly secreted in drug resistant cancer cells. The pharmacological inhibition of NF-κB, PI3-K/Akt and MEK/Erk and the pharmacological inhibition and genetic inhibition (Stat3 siRNA) of Jak2/Stat3 pathway decreased IL-6 autocrine production in various drug resistant cancer cell lines and similarly decreased IL-6 autocrine production in clinically isolated lung cancer cells.

**Conclusions:**

This study is the first to directly address the role Stat3 plays on the autocrine production of IL-6, which occurs through a positive-feedback loop. Our biochemical and genetic studies clearly demonstrated that Jak2/Stat3, in combination with other IL-6 downstream pathways, contributed frequently and substantially to IL-6 autocrine production in a broad spectrum of cancer cell lines as well as in clinical cancer samples. Our findings suggest that Stat3 could potentially be regulated to suppress IL-6 autocrine production in cancer cells to inhibit the progression of cancer and reduce drug resistance.

## Background

Interleukin-6 (IL-6) is a multifunctional cytokine that normally modulates a variety of physiological events including cell survival and apoptosis [1], but its dis-regulation has been implicated in various diseases including cancer [2-4] for which it has been associated with tumor progression, drug resistance and poor prognosis [5-7]. IL-6 signaling is triggered by the binding of IL-6 to its specific ligand-binding subunit of the receptor (gp80) to induce phosphorylation and homodimerization of the common signaling-subunit of the receptor (gp130). Three major downstream signaling cascades are then activated: MEK/extracellular signal-related kinase (Erk), phosphatidylinositol 3-kinase (PI3-K)/Akt and Janus kinase (Jak) 2/signal transducer and activator of transcription 3 (Stat3) [8]. These cascades, the most well-known being Jak2/Stat3 cascade, are responsible for IL-6 mediated cellular responses for both the physiological and pathological events [9].

Like all members of the Stat family proteins, Stat3 is a latent cytoplasmic transcription factor activated in response to growth factors and cytokines through the phosphorylation of a single tyrosine residue [9]. This phoshorylation is usually an indicator that Stat3 has been activated. Activated Stat3 forms a dimer and translocates to the nucleus where it binds to DNA in the promoter region of target genes to regulate gene transcription. It has been previously found that the functioning of endogenous Stat3 was inhibited when cells were transfected with S3F (a dominant-negative Stat3 mutant that cannot be tyrosine phosphorylated) or S3D (a dominant-negative Stat3 mutant that cannot bind to DNA) while an additional functioning of exogenous Stat3 was supplied when cells are transfected with S3C (a constitutively-active Stat3 mutant forced to form a dimer constitutively without stimulation) [2,10]. The ability of these mutants to affect the functioning of Stat3 makes it possible to study the effect of Stat3 on gene regulation.

IL-6 is induced by a variety of stimuli that mostly achieve this through their activation of NF-κB, C/EBP, CREB and AP-1, which are transcription factors known to bind to IL-6 promoter [11-13]. IL-6 is also known to be auto-regulated in many types of cells [14,15]. For example, MEK/Erk and PI3-K/Akt, which are, as mentioned above, downstream pathways triggered by IL-6, also work upstream to regulate the expression of IL-6. PI3-K/Akt does this by activating IKK-α which in turn activates AP-1 and NF-κB to induce the expression of IL-6 [16,17], and MEK/Erk kinase does this by activating NF-κB [18]. Recently, NF-κB, long known to be an important upstream regulator of IL-6 expression [12,13], has been found to be activated downstream by IL-6 [19]. However, the role of the most well-known IL-6 downstream signal, Jak2/Stat3 pathway, remains controversial.

Some studies have suggested that Jak2/Stat3 pathway may also be involved upstream in the regulation of IL-6, but other studies disagree. Studies not directly investigating the role of Stat3 on the expression of IL-6 in cancer cells have found some evidence suggesting Stat3 may increase IL-6 expression. IL-6 mRNA was found to be elevated in tumor tissue in gp130 mutant mice with abnormally activated Stat3 [20]. IL-6 mRNA was found to be up-regulated in alveolar type II epithelial cells of transgenic mice over-expressing S3C in a tissue-specific manner [21]. In more recent studies of the role of Stat3 in immune responses in macrophages and fibroblasts, Ogura *et al*. reported that IL-6 as well as other cytokines could be decreased by inhibiting Stat3 [22-24]. Another study investigating the role of Stat3 in immune evasion in human melanoma cells, has reported that Stat3 siRNA decreased the mRNA expression of IL-6, IL-10 and VEGF [25]. Gao *et al*. showed that mutant EGFR could activate the gp130/Jak/Stat3 pathway to increase tumorigenesis by up-regulation of IL-6 but the authors did not specifically knock-down Stat3 to show the increase of IL-6 secretion by mutant EGFR is mediated by Stat3 activation in their study [26]. However, two immunological studies investigating the effect of over-expression of S3C on the production of cytokines found that transfection of S3C suppressed the expression of IL-6 in macrophages [27,28]. Another important study investigating the possible effect of Stat3 on immune suppression of cancer cells found that the inhibition of Stat3 with antisense oligonucleotide and with dominant-negative form of Stat3 (Stat3β) resulted in an increase in IL-6 in mouse cancer cells [29]. Because these investigations were not designed specifically to study or to provide direct evidence of the role of Stat3 on the expression IL-6 in cancer cells, we performed biochemical and genetic studies of manipulating the Stat3 function to clarify its role on the autocrine production of IL-6 in various cancer cell lines and human tumor samples.

## Methods

### Materials

The AG490, LY294002, U0126, BAY11-7082 and PD98059 inhibitors were purchased from Biomol (Plymouth, PA, USA). The chemotherapeutic agents, paclitaxel, camptothecin, vincristine and etoposide were purchased from Sigma (St Louis, MO, USA). Epirubicin was purchased from Merck (Darmstadt, Germany).

### Cell culture

For this study, we used one human lung adenocarcinoma cell line PC14PE6/AS2 (AS2) to study the effect of IL-6 downstream pathways on IL-6 autocrine production and drug resistance. We had previously established this cell line and found it to produce autocrine IL-6 which activated Stat3 and subsequently promoted tumor progression [2]. In addition, we used a series of AS2-derived cell lines: one vector cell line (AS2/Vec-11) and 6 mutant cell lines expressing plasmids containing constitutively-active (AS2/S3C cells: AS2/S3C-A and AS2/S3C-C) or dominant-negative Stat3 (AS2/S3D cells: AS2/S3D-8, AS2/S3D-9, and AS2/S3F cells: AS2/S3F-3, and AS2/S3F-7).

We used 3 other cancer cell lines, MCF-7, KB and A549 (American Type Culture Collection) and their derived drug resistant cell lines. The MCF-7 derived drug resistant cell line MCF-7/ADR was kindly provided by Dr. Chih-Hsin Yang (National Taiwan University, Taipei, Taiwan). This cell line was maintained with 1 μM epirubicin to ensure it retained its drug resistance [30]. We used 5 other drug resistant cell lines that we had previously established from KB and A549 cells: KB-CPT100 maintained with 100 nM camptothecin; KB-TAX50 maintained with 50 nM paclitaxel; KB-VIN10 maintained with 10 nM vincristine; KB-7D maintained with 1 μM etoposide; and A549-T12 maintained with 12 nM paclitaxel [31,32].

AS2- and MCF-7 parental and derived cells were maintained in MEM-α and DMEM medium (Invitrogen, Carlsbad, CA, USA), respectively, with 10% fetal calf serum (FCS; Invitrogen), and KB and A549 parental and derived cells were maintained in RPMI 1640 (Invitrogen) with 5% FCS.

### Patient and sample processing

Lung cancer cells were collected from the lung cancer associated malignant pleural effusion (MPE) of twenty patients treated at National Cheng Kung University Hospital. Each patient provided written informed consent. Each sample was verified to be positive by cytological analysis of MPE or pathological proof based on a pleural biopsy. MPE samples were collected and centrifuged immediately. Tumor cells were separated from MPE-associated lymphocytes by serial gradient centrifugation with Histopaque1077 and Percoll (Sigma) as previously described [33]. The purity of tumor cells was determined by cytological analysis to be between 70% and 90%. Frozen samples were cryopreserved in 90% FCS/10% DMSO. Freshly isolated or defrosted cells were suspended in RPMI 1640 medium with 10% FCS and allowed to rest at 37°C for 1 hour before treatment with signal pathway inhibitors. The protocol for this study was approved the institutional review board at National Cheng Kung University Hospital.

### Enzyme-linked immunosorbent assay (ELISA) for IL-6

Attached cells were plated at concentrations of 0.5 × 10^5 ^- 3 × 10^5 ^cells/ml/well in 12-well plates. The suspended cancer cells obtained from MPE were grown in sterile tubes to a concentration of 2.5 × 10^5 ^cells/ml/tube. After treatment, the conditioned media were collected at indicated time points and stored at -20°C until further use. The collected samples were assayed using a commercially available ELISA kit (Invitrogen).

### Cell lysis and Western blot analysis

For cell lysis, the harvested cells were incubated on ice in whole-cell-extract lysis buffer for 30 min, lysates were centrifuged at 14000 rpm for 10 min, and protein concentration measured by Bradford assay (Bio-Rad, Richmond, CA, USA). For Western blot analysis, lysates were then boiled for 5 min with sample buffer before being separated on SDS-polyacrylamide gels. Proteins were transferred to polyvinylidene difluoride membranes (Millipore, Billerica, MA, USA) and blocked with 5% nonfat milk/TBST buffer. Using an electrochemiluminescence kit (Amersham Pharmacia, Biotech Inc., Piscataway, NJ, USA), we detected binding of eight specific antibodies: (1) anti-phospho-Stat3 (Tyr705) (Cell Signaling, Danvers, MA, USA), (2) anti-Stat3 (BD Biosciences, San Jose, CA, USA), and (3) anti-actin (Millipore) (4) anti-phospho-Akt (Ser473) (Cell Signaling), (5) anti-Akt (Cell Signaling), (6) anti-Akt1 (Cell Signaling), (7) anti-phospho-Erk (R & D, Minneapolis, MN, USA), and (8) anti-Erk (Santa Cruz Biotechnology, Santa Cruz, CA, USA).

### MTT assay

Cells were seeded at concentrations of 5×10^3 ^- 7.5×10^3 ^cells/200 μl/well in 96-well plates. After treatment, one-tenth of the original culture volume of MTT (Sigma) stock solution was added to the wells and incubated for 4 hours at 37°C. After removing the supernatant by centrifugation, DMSO was added to release MTT.

### Luciferase reporter assays

The p1168huIL6P-luc+, a pGL3 based IL-6 promoter luciferase reporter plasmid containing 1168 bp of the human IL-6 promoter, was kindly provided by Dr. Hsiao-Sheng Liu (National Cheng Kung University, Tainan, Taiwan) [34], the mammalian expression plasmid for the dominant-negative mutant of Stat3 (S3D) by Dr T Hirano [35], and the active-form Stat3 (SC) plasmid by Dr James Darnell Jr [36]. The p1168huIL6P-luc+ plasmid, the control phRL-TK plasmid (for normalization), and either MQ water (mock) or control vector or S3C plasmid or S3D plasmid were co-transfected into AS2 cells using MicroPorator MP-100 (NanoEnTek, Seoul, South Korea). Firefly and Renilla luciferase activities were then measured in cell extracts using the Dual-Luciferase Reporter Assay System (Promega, MI, USA). Data were presented as the ratio of Firefly luciferase activity to Renilla luciferase activity, and normalized with the control group.

### RNA extraction and semiquantitative RT-PCR

Total RNA was extracted using the single-step TRIzol method (Invitrogen). For RT-PCR, the first-strand cDNA was synthesized from 0.2 μg of total RNA with oligo-dT primer and the AMV reverse transcriptase (Promega, Madison, WI, USA). The sequences of PCR primers were: IL-6 sense, 5'-CTTTTGGAGTTTGAGGTAGTATACCTA-3'; IL-6 antisense, 5'-GCTGCGCAGAATGAGATGAGTTGTC-3'; β-actin sense, 5'-AGCGGGAAATCG TGCGTG-3' and β-actin antisense, 5'-CAGGGTACATGGTGGTGGTGCC-3'. PCR was performed as follows: after incubation at 94°C for 5 min, IL-6 underwent 30 cycles and β-actin 17 cycles of reaction (94°C for 30 sec, 52°C for 30 sec and 72°C for 1 min). After cycling, the samples were incubated at 72°C for 10 min.

### siRNA, shRNA and transfection

To knock-down Stat3, Akt1, Erk1 and Erk2 we used synthetic siRNAs with different targeting sequences: Stat3#1, Stat3#2, Akt1, Erk1 and Erk2 (Ambion, Austin, TX, USA). A scramble siRNA was used as a negative control (Invitrogen). Cells were transfected with siRNA to a final concentration of 50 or 100 nM with MicroPorator MP-100 (NanoEnTek). For long-term suppression of Stat3 expression, Stat3#1 sequence was cloned into the pSUPER vector, kindly provided by Dr R. Agami, The Netherlands Cancer Institute, Amsterdam, Netherlands, as previously described [37,38]. Cells were transfected with shRNA using MicroPorator MP-100. After transfection, we treated the cells with Hygromycin-B (Invitrogen) for more than 3 weeks to select stable cell lines containing Stat3 shRNA or control plasmid. The stable cell lines were maintained in media containing 300 μM Hygromycin-B and passaged once in the absence of Hygromycin-B before treatment.

### Statistical analysis

Results were expressed as the mean ± standard error of the mean. Statistical significance was set at P < 0.05. Differences between two independent groups were determined using the Student t-test. Differences between two paired groups were determined using paired t-test. All statistical operations were performed using Prism4 (GraphPad Software, San Diego, CA, USA).

## Results

### Autocrine IL-6 induced Stat3 activation and paclitaxel resistance in AS2 cells

**Figure 1:**
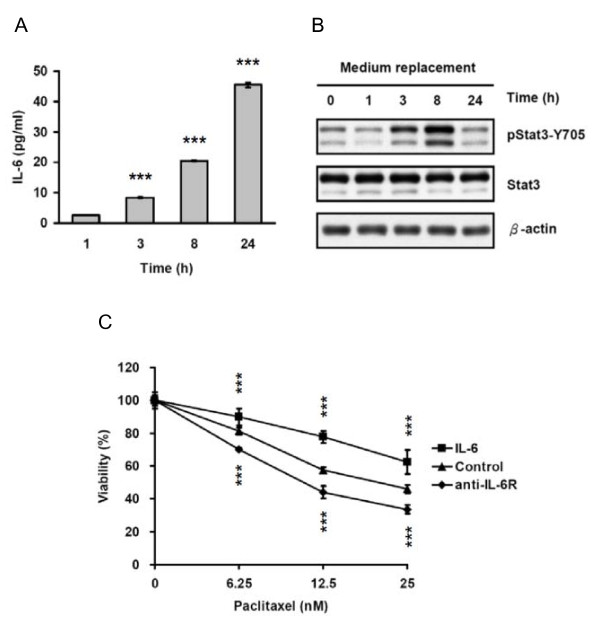
**Autocrine IL-6 induced Stat3 activation and paclitaxel resistance in AS2 cells**. (A) AS2 cells constitutively secreted IL-6. AS2 cells were seeded for 24 hours before medium replacement. Culture supernatants were collected 1, 3, 8, or 24 hours after medium replacement and the IL-6 levels were detected by ELISA. This graph shows the results as mean ± SEM. Student's t tests, ***p < 0.001. (B) Autocrine IL-6 induced Stat3 activation in AS2 cells. AS2 cells were seeded for 24 hours before medium replacement. Cell lysates were collected 1, 3, 8, or 24 hours after medium replacement. The activation of Stat3 was evaluated by Western blot analysis. (C) Autocrine IL-6 rendered AS2 cells resistant to paclitaxel. AS2 cells were seeded for 24 hours and then treated as following: (1) cells without pretreatment and treated with varying doses of the chemotherapeutic agent palitaxel only for 72 hours, (2) cells pretreated with exogenous IL-6 (20 ng/ml) for 1 hour and then cotreated with exogenous IL-6 + paclitaxel for 72 hours, and (3) cells pretreated with anti-IL-6 receptor neutralizing antibody (anti-IL-6R) (5 μg/ml) for 1 hour and then cotreated with anti-IL-6R + paclitaxel for 72 hours. The viability of AS2 cells was analyzed by MTT assay. This graph shows the results as mean ± SEM. Student's t tests, ***p < 0.001.

We previously demonstrated that AS2 cells produced autocrine IL-6 and the secreted IL-6 induced Stat3 activation and subsequently promoted tumor progression [2]. We used ELISA and Western blot analysis to measure IL-6 secretion and Stat3 activation (phosphorylation) after medium replacement to remove the existing IL-6 in the old medium and make it possible to measure the amount of newly secreted IL-6 time-dependently in AS2 cells, respectively. We found the constitutive secretion of IL-6 at hours 1 to 24 and the activation of Stat3 peaked at hours 3 and 8, confirming the autocrine production of IL-6 and the subsequent activation of Stat3 in AS2 cells (Figure 1A and 1B).

It has been shown that cancer cells resistant to chemotherapeutic agents express elevated levels of IL-6, and the IL-6 contributes to the drug resistance of cancer cells [30,39]. In our MTT assay of the effect of IL-6 on paclitaxel sensitivity in AS2 cells, we found a significant increase (about 15%) in cell viability in cells pre-treated with exogenous IL-6 and a significant decrease (about 15%) in cell viability in cells treated with anti-IL-6R, compared to the un-pretreated cells (both p < 0.001), indicating that autocrine IL-6 contributed to the paclitaxel resistance in AS2 cells (Figure 1C).

### Jak2/Stat3 pathway positively regulated IL-6 autocrine production in AS2 cells

**Figure 2:**
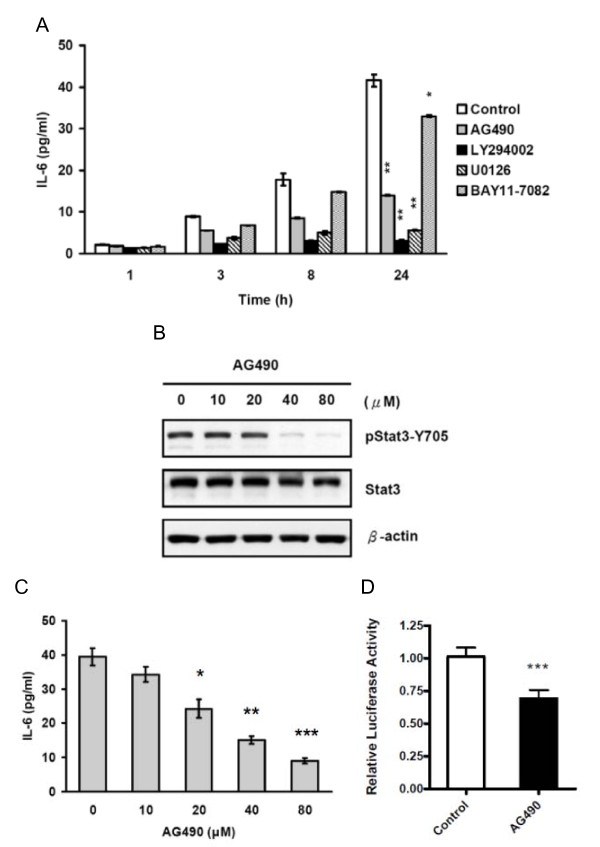
**Jak2/Stat3 pathway positively regulated IL-6 autocrine production in AS2 cells**. (A) Pharmacological inhibition of Jak2/Stat3, PI3-K/Akt, MEK/Erk and NF-κB pathways decreased the secretion of IL-6. AS2 cells were seeded for 24 hours and then treated with or without the Jak2/Stat3 inhibitor (AG490, 40 μM), the PI3-K/Akt inhibitor (LY294002, 20 μM), the MEK/Erk inhibitor (U0126, 5 μM), or the NF-κB inhibitor (BAY11-7082, 20 μM) for 1, 3, 8, or 24 hours. The culture supernatants were collected at the indicated time points and analyzed by ELISA. This graph shows the results as mean ± SEM. Student's t tests, *p < 0.05 and **p < 0.01. For a clearer demonstration, statistical significances are shown for the 24-hour time points only. (B and C) AG490 inhibited Stat3 phosphorylation and IL-6 secretion in a dose-dependent manner. AS2 cells were seeded for 24 hours and then treated with the indicated doses of AG490 for 24 hours. Its effects on Stat3 phosphorylation were analyzed by Western blot analysis (B) and IL-6 secretion by ELISA (C). The graph (C) shows the results as mean ± SEM. Student's t tests, *p < 0.05, **p < 0.01, and ***p < 0.001. (D) AG490 decreased IL-6 promoter luciferase activity. AS2 cells were co-transfected with the p1168huIL6P-luc+ IL-6 reporter plasmid and the control phRL-TK plasmid. The cells were incubated for 24 hours and then treated with or without AG490 (40 μM) for 24 hours. The cells were lysed and subjected to Firefly and Renilla luciferase activity measurements. This graph shows the results as mean ± SEM. Student's t tests, ***p < 0.001.

To investigate whether Jak2/Stat3 as well as the other three IL-6 downstream pathways (PI3-K/Akt, MEK/Erk, and NF-κB) known to be involved in IL-6 expression in various cells would act as an upstream regulator of IL-6 autocrine production in AS2 cells, we used ELISA to measure IL-6 secretion in one control AS2 group and in four different AS2 treatment groups each with one pathway (Jak2/Stat3, PI3-K/Akt, MEK/Erk, or NF-κB) pharmacologically inhibited by the inhibitors AG490, LY294002, U0126, or BAY11-7082, respectively. We found that, compared to the controls, MEK/Erk inhibitor and PI3-K/Akt inhibitor reduced IL-6 secretion in AS2 cells by about 80% and 90% (both p < 0.01), but NF-κB inhibitor decreased it by only 20% (p < 0.05) (Figure 2A). Importantly, Jak2/Stat3 inhibitor also reduced IL-6 secretion by more than 60% (p < 0.01). Though Jak2/Stat3 inhibitor was not the most efficient, Jak2/Stat3 pathway clearly participates in the regulation of IL-6 and should be significant an upstream regulator of IL-6 secretion in AS2 cells (Figure 2A). To exclude the possibility that the reduction of IL-6 secretion was mainly caused by the reduction of cell survival, cell viability was measured by MTT assay after being treated with each one of four inhibitors. None of these inhibitors compromised the viability of AS2 cells during the treatment period at the indicated doses (Additional file 1, Figure S1A).

To confirm our findings, we performed inhibition experiments on AS2 cells using increasing doses of Jak2/Stat3 inhibitor. Decrease in Stat3 phosphorylation was confirmed by Western blot analysis, and IL-6 secretion was measured by ELISA. We found the Jak2/Stat3 inhibitor dose-dependently decreased Stat3 phosphorylation (Figure 2B) and IL-6 secretion (p < 0.05 at doses higher than 20 μM) (Figure 2C). We also used MTT assay to analyze the effect of the increasing doses of AG490 on cell viability and showed that only a minor reduction in cell survival (about 15%) was found when cells exposed to 80 μM AG490 (Additional file 1, Figure S1B). In addition, we showed that treatment with AG490 (40 μM) significantly decreased IL-6 promoter activity (Figure 2D). Our results suggest that Jak2/Stat3 pathway may regulate the autocrine production of IL-6 in AS2 cells.

### Stat3 activation status was positively correlated with IL-6 expression and paclitaxel resistance in AS2-derived cells

**Figure 3:**
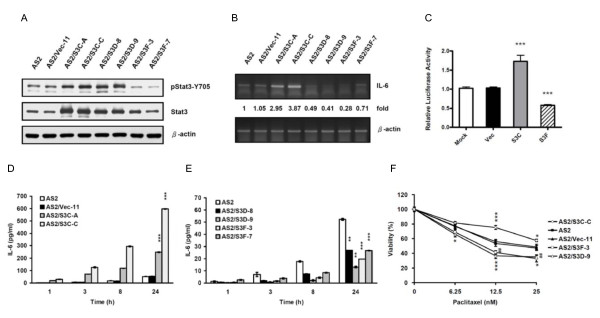
**Stat3 activation status was positively correlated with IL-6 expression and paclitaxel resistance in AS2-derived cells**. (A) AS2-derived cells expressed various Stat3 mutant proteins. Cell lysates were collected 24 hours after seeding. The total amount of Stat3 protein and Stat3 phosphorylation level (pStat3-Y705) were analyzed by Western blot analysis. (B) Stat3 activation status positively correlated with IL-6 mRNA expression. RNA samples were collected 24 hours after seeding. The expression of IL-6 mRNA was analyzed by RT-PCR. IL-6 mRNA levels of each cell were normalized to β-actin mRNA levels and indicated as a relative fold to that of the parental AS2 cell. (C) Stat3 positively regulated IL-6 promoter luciferase activity. The p1168huIL6P-luc+ IL-6 reporter plasmid and the control phRL-TK plasmid were co-transfected without (mock) or with control vector (Vec) or S3C plasmid or S3D plasmid into AS2 cells. The cells were incubated for 24 hours and then lysed and subjected to Firefly and Renilla luciferase activity measurements. This graph shows the results as mean ± SEM. Student's t tests, ***p < 0.001. (D and E) Stat3 activation status positively correlated with IL-6 secretion. The cells were seeded for 24 hours before medium replacement and the culture supernatants were collected 1, 3, 8, or 24 hours after medium replacement. IL-6 secretion was analyzed by ELISA. The graphs show the results as mean ± SEM. Student's t tests, **p < 0.01 and ***p < 0.001. For a clearer demonstration, statistical significances are shown for the 24-hour time points only. (F) Stat3 activation status positively correlated with paclitaxel resistance. Cells were seeded for 24 hours and then treated with paclitaxel at the indicated doses for another 72 hours. Cell viability was determined by MTT assay. The graph represents the results as mean ± SEM. Student's t tests, *p < 0.05, ## p < 0.01 and ***p < 0.001 (For a clearer demonstration, the p of AS2/S3F-3 were shown as #.).

To clarify the role of Stat3 on IL-6 autocrine production proposed by the biochemical studies, we performed genetic studies to investigate the effect of varying degrees of Stat3 activation and inactivation on the mRNA expression and the secretion of IL-6 using parental AS2 cells and various previously established AS2-derived cell lines with different Stat3 activation status: vector control cells, two AS2/S3C cells, two AS2/S3D cells, and two AS2/S3F cells [2,10]. In this current study, we used S3C as active form Stat3, and S3D and S3F as inactivated forms of Stat3. Western blot analysis showed increased expression of Stat3 protein in all mutant cells, in the AS2/S3C cells (AS2/S3C-A and AS2/S3C-C), in the AS2/S3D cells (AS2/S3D-8 and AS2/S3D-9) and in the AS2/S3F cells (AS2/S3F-3 and AS2/S3F-7), compared to the parental cells (AS2) and vector control cells (AS2/Vec-11) (Figure 3A). However, only AS2/S3F cells but not AS2/S3C or AS2/S3D cells were found to have decreases in Stat3 phosphorylation (Figure 3A). RT-PCR showed that the AS2/S3C cells expressed 3 to 4 times more IL-6 mRNA than the parental and vector control cells and that AS2/S3D and AS2/S3F cells expressed 30 to 70 percent less IL-6 mRNA (Figure 3B). Similarly, transient transfection with S3C plasmid increased IL-6 promoter luciferase activity by more than 70% and transient transfection with S3F plasmid decreased IL-6 promoter luciferase activity by more than 40% compared with the mock and vector control groups (both p < 0.001) (Figure 3C). ELISA showed that AS2/S3C cells secreted 5 to 10 times more IL-6 than the parental and vector control cells (both p < 0.001) (Figure 3D) and AS2/S3D and AS2/S3F cells secreted 40 to 80 percent less IL-6 (both p < 0.01 in AS2/S3D cells and both p < 0.001 in S3F cells) (Figure 3E). These results show that Stat3 may positively regulate the expression of IL-6 mRNA expression and the secretion of IL-6 in AS2 cells.

To evaluate drug resistance, we treated the parental AS2 cells, vector control cells (AS2/Vec-11), the AS2/S3C cells (AS2/S3C-C), the AS2/S3D cells (AS2/S3D-9), and the AS2/S3F cells (AS2/S3F-3) with paclitaxel for 72 hours. Using MTT assay to access cell viability, we found AS2 cells with increased Stat3 activity (AS2/S3C-C) to be more resistant to paclitaxel than AS2 and AS2/Vec-11 cells (p < 0.05), and AS2 cells with decreased Stat3 activity (AS2/S3D-9 and AS2/S3F-3) to be less resistant to paclitaxel (p < 0.05) (Figure 3F). Together, these findings suggest that the activation of Stat3 may contribute to the regulation of IL-6 autocrine production and resistance to paclitaxel in AS2 cells.

### Knocking-down Stat3 by transient transfection with synthetic siRNA decreased IL-6 expression in AS2 cells

**Figure 4:**
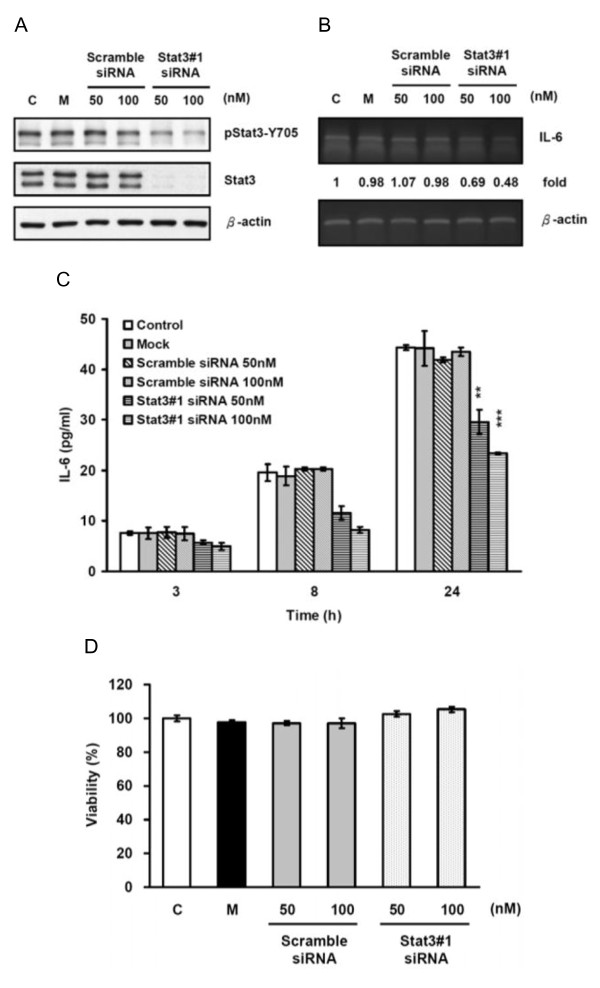
**Knocking-down Stat3 by transient transfection with synthetic siRNA decreased IL-6 expression in AS2 cells**. (A) Transient transfection with synthetic Stat3 siRNA effectively knocked-down Stat3 expression. AS2 cells were left untreated as controls (C), transfected with nothing as mocks (M), or transfected with two different doses of scramble control siRNA or Stat3 siRNA (Stat3#1). The cells were incubated for 72 hours and then cell lysates were collected. The total amount of Stat3 protein and Stat3 phosphorylation level (pStat3-Y705) were analyzed by Western blot analysis. (B) Transient transfection with Stat3 siRNA decreased IL-6 mRNA expression. 72 hours after transfection, the medium was replaced and RNA samples were collected 24 hours afterwards. IL-6 mRNA expression was measured by RT-PCR. IL-6 mRNA levels of each sample was normalized to β-actin mRNA levels and indicated as a relative fold to that of the control. (C) Transient transfection with Stat3 siRNA decreased IL-6 secretion. 72 hours after transfection, the medium was replaced and culture supernatants were collected 3, 8 and 24 hours afterwards. IL-6 secretion was measured by ELISA. The graph represents the results as mean ± SEM. Student's t tests, **p < 0.01 and ***p < 0.001. For a clearer demonstration, statistical significances are shown for the 24-hour time points only. (D) Knocked-down Stat3 did not affect cell viability. The viability of cells was determined by MTT assay 72 hours after transfection. The graph represents the results as mean ± SEM.

To confirm that Stat3 regulated IL-6 expression in cancer cells, we transiently transfected AS2 cells with Stat3 siRNA to knock-down the expression of Stat3. Western blot analysis showed transfection with Stat3 siRNA (Stat3#1) dose-dependently decreased the total amount of Stat3 protein and phosphorylated Stat3 (Figure 4A). RT-PCR and ELISA showed transfection with Stat3#1 reduced the expression of IL-6 mRNA (Figure 4B) and the secretion of IL-6 at 3, 8, and 24 hours after medium replacement (p < 0.01 in the lower dose and p < 0.001 in the higher doses) (Figure 4C). To make sure our results were not confounded by differences in cell viability, we performed MTT assay of the transfected and untransfected cells, and found that these siRNAs did not affect the viability of AS2 cells (Figure 4D). The findings suggested that the suppression of IL-6 production by knocking-down Stat3 was not likely a result of a decrease in cell number. As can be seen in Figures S2A and S2B in Additional file 2, the other Stat3 siRNA (Stat3#2) with a different targeting sequence also knocked-down Stat3 expression and reduced IL-6 secretion but did not compromise cell proliferation (Additional file 3, Figure S3A), a further confirmation of our findings.

### Knocking-down Stat3 by stable transfection with shRNA decreased the expression of IL-6 in AS2 cells

**Figure 5:**
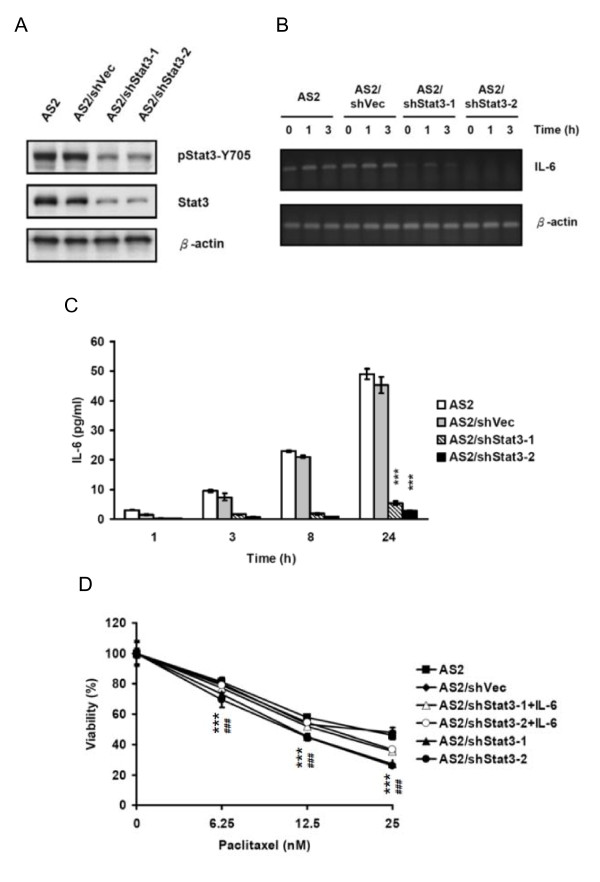
**Knocking-down Stat3 by stable transfection with shRNA decreased the expression of IL-6 in AS2 cells**. (A) Stable cell lines were transfected with plasmid containing Stat3 shRNA (AS2/shStat3-1 and AS2/shStat3-2) or control plasmid (AS2/shVec) and selected with 300 μM Hygromycin-B. The cell lysates were collected 24 hours after seeding. The total amount of Stat3 protein and Stat3 phosphorylation level (pStat3-Y705) were analyzed by Western blot analysis. (B) The mRNA expression of IL-6 was decreased in stable cell lines expressing Stat3 shRNA. The cells were seeded 24 hours before medium replacement. RNA samples were collected 0, 1 and 3 hours after medium replacement and IL-6 mRNA expression was measured by RT-PCR. (C) The secretion of IL-6 was decreased in stable cell lines expressing Stat3 shRNA. The cells were seeded 24 hours before medium replacement. Culture supernatants were collected 1, 3, 8, or 24 hours after medium replacement. IL-6 secretion was measured by ELISA. The graph represents the results as mean ± SEM. Student's t tests, ***p < 0.001. For a clearer demonstration, statistical significances are shown for the 24-hour time points only. (D) Stat3 shRNA expressing cells showed lower resistance to paclitaxel and pretreatment with exogenous IL-6 modestly restored the resistance. Cells were seeded for 24 hours and then treated as following: (1) cells without pretreatment and treated with varying doses of the chemotherapeutic agent paclitaxel only for 72 hours, and (2) cells pretreated with exogenous IL-6 (20 ng/ml) for 1 hour and then cotreated with exogenous IL-6 + paclitaxel for 72 hours. The viability of cells was analyzed by MTT assay. The graph represents the results as mean ± SEM. Student's t tests, ***p < 0.001 and ###p < 0.001 (For a clearer demonstration, the p of AS2/shStat3-2 were shown as #.). For a clear demonstration, statistical significances between the IL-6 pre-treated and un-pretreated groups were not shown.

To further investigate the possible role of Stat3 in the regulation of IL-6, we stably transfected AS2 cells with the control vector from which we selected one cell line (AS2/shVec) and the vector expressing Stat3 shRNA from which we selected two cell lines (AS2/shStat3-1 and AS2/shStat3-2). Western blot analysis showed a lower expression of Stat3 protein and a lower level of Stat3 phosphorylation in both cell lines expressing Stat3 shRNA than in either the parental cells or the vector control cells (Figure 5A). RT-PCR showed a continuing decrease in the expression of IL-6 mRNA in both cell lines expressing Stat3 shRNA (Figure 5B). ELISA also showed a continuing decrease IL-6 secretion in both cell lines expressing Stat3 shRNA compared to the parental AS2 (90% in AS2/shStat3-1 and 95% AS2/shStat3-2 at 24 hours) (p < 0.001) (Figure 5C).

We also analyzed the drug resistance of these cells to paclitaxel by MTT assay. MTT assay showed that the permanent knock-down of Stat3 in AS2/shStat3-1 and AS2/shStat3-2 cells significantly reduced their resistance to paclitaxel (p < 0.001) (Figure 5D). Pretreatment with exogenous IL-6 modestly restored the resistance (p < 0.01) (Figure 5D). These data suggest that the IL-6-induced paclitaxel resistance is mediated by both Stat3-dependent and Stat3-independent pathways.

### Stat3 contributed to the elevation of IL-6 in drug resistant cancer cells

**Figure 6:**
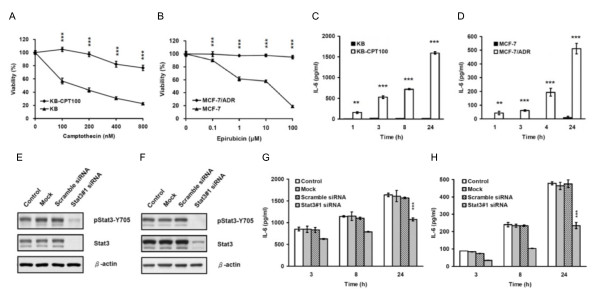
**Stat3 contributed to the elevation of IL-6 in drug resistant cancer cells**. (A and B) KB-CPT100 and MCF-7/ADR cells exhibited higher drug resistance than their parental KB and MCF-7 cells. The cells were seeded for 24 hours and treated with camptothecin (KB-CPT100 and KB cells) or epirubicin (MCF-7/ADR and MCF-7 cells) at the indicated doses for another 72 hours. The viability of cells was determined by MTT assay. (C and D) IL-6 expression was elevated in the two drug resistant cancer cells: KB-CPT100 and MCF-7/ADR compared to their parental cells. The cells were seeded 24 hours before medium replacement. Culture supernatants were collected 1, 3, 8, or 24 hours after medium replacement. IL-6 secretion was determined by ELISA. The graphs (A-D) represent the results as mean ± SEM. Student's t tests, **p < 0.01 and ***p < 0.001. (E and F) Transient transfection with Stat3 siRNA effectively knocked-down Stat3 in these two drug resistant cells. Cells were left untreated as controls (C), transfected with nothing as mocks (M), or transfected with 50 nM of scramble control siRNA or Stat3 siRNA (Stat3#1). The cells were incubated for 72 hours and then cell lysates were collected. The total amount of Stat3 protein and Stat3 phosphorylation level (pStat3-Y705) were analyzed by Western blot analysis. (G and H) IL-6 secretion was suppressed by transient transfection with Stat3 siRNA in these two drug resistant cells. Medium replacement was performed 72 hours after transfection. Culture supernatants were collected 3, 8, or 24 hours after medium replacement and IL-6 secretion was determined by ELISA. The two graphs represent the results as mean ± SEM. Student's t tests, ***p < 0.001. For a clearer demonstration, statistical significances are shown for the 24-hour time points only.

It has been shown that cancer cells resistant to chemotherapeutic agents express elevated levels of IL-6 [30,39]. Thus, drug resistant cancer cells are ideal models for studying IL-6 autocrine production. To find out whether IL-6 would be regulated by Stat3 in cancer cell lines other than AS2, we performed genetic siRNA experiments on two drug resistant cancer cell lines (KB-CPT100 and MCF-7/ADR). MTT assay revealed that KB-CPT100 cells were more resistant to the chemotherapeutic agent camptothecin than the parental KB cells (Figure 6A) and MCF-7/ADR cells were much more resistant to the chemotherapeutic agent epirubicin than the parental MCF-7 cells (p < 0.001) (Figure 6B). The two drug resistant cell lines were found by ELISA to secrete more IL-6 than their parental cells (Figures 6C and 6D).

We transiently transfected the two drug resistant cells with Stat3#1 to knock-down Stat3. Western blot analysis confirmed that the total amount of Stat3 protein and phosphorylated Stat3 had been knocked-down in both resistant cells (Figures 6E and 6F). MTT assay found no change in cell viability (Additional file 3, Figure S3B and S3C). ELISA revealed that knocking-down Stat3 decreased the secretion of IL-6 in KB-CPT100 cells by one-third (p < 0.001) (Figure 6G) and by one-half in MCF-7/ADR cells (p < 0.001) (Figure 6H). These results suggest that the Stat3 also contributes to the elevation of IL-6 in drug resistant cancer cells.

### Jak2/Stat3 pathway regulated the expression of IL-6 in cooperation with other IL-6 downstream pathways

**Figure 7:**
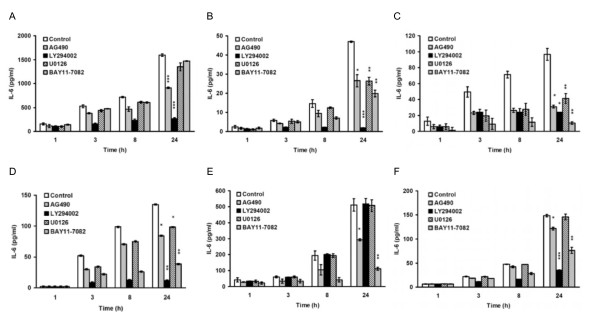
**Jak2/Stat3 pathway regulated the expression of IL-6 in cooperation with other IL-6 downstream pathways**. (A-F) Pharmacological inhibition of Jak2/Stat3, PI3-K/Akt, MEK/Erk and NF-κB pathways decreased the secretion of IL-6 in drug resistant cancer cells. Of the six drug resistant cancer cells used in this experiment, four were KB-derived, (A) to (D), respectively: KB-CPT100 is resistant to camptothecin; KB-TAX50 to paclitaxel, KB-VIN10 to vincristine and KB-7D to etoposide. The other two were MCF-7-derived multidrug resistant MCF-7/ADR cells (E) and A549-derived paclitaxel resistant A549-T12 cells (F). All cells were seeded for 24 hours and then treated with AG490 (40 μM), LY294002 (20 μM), U0126 (5 μM), BAY11-7082 (20 μM), or medium alone for 1, 3, 8, or 24 hours. The culture supernatants were collected at the indicated time points and IL-6 secretion was measured by ELISA. The graphs (A-F) represent the results as mean ± SEM. Student's t test: *p < 0.05, **p < 0.01, and ***p < 0.001. For a clearer demonstration, statistical significances are shown for the 24-hour time points only.

To find out whether IL-6 could be regulated by different combinations of its downstream pathways including Jak2/Stat3 in various cancer cells, we pharmacologically inhibited the four IL-6 downstream pathways in six drug resistant cancer cell lines derived from cervical cancer, breast cancer, and lung cancer cells. ELISA revealed that all drug resistant cells secreted more IL-6 than their parental cells. Different cells used different combinations of signaling pathways, including Jak2/Stat3, to regulate secretion of IL-6 (Figures 7A to 7F). To exclude the possibility that the reduction of IL-6 secretion was caused by the reduction of cell survival, we used MTT assay to analyze the effect of these inhibitors on cell viability (Additional file 1, Figure S1C to S1H). We showed that the majority of inhibitors had only limited suppressive effect on cell viability (below 25%) (Additional file 1, Figure S1C-S1H) except that the PI3-K/Akt pathway inhibitor LY294002 had more suppressive activity on the cellular viability by 30 to 50% (Additional file 1, Figure S1C-S1F). However, LY294002 induced much greater (75 to 90%) decrease of IL-6 in these cells (Figure 7A to 7D). There is only one exception that the AG490-induced reductions of cell survival and IL-6 secretion were both about 30% in KB-7D cells (Additional file 1, Figure S1F and Figure 7D).

### Jak2/Stat3 pathway contributed to IL-6 autocrine production in clinically isolated lung cancer cells

**Figure 8:**
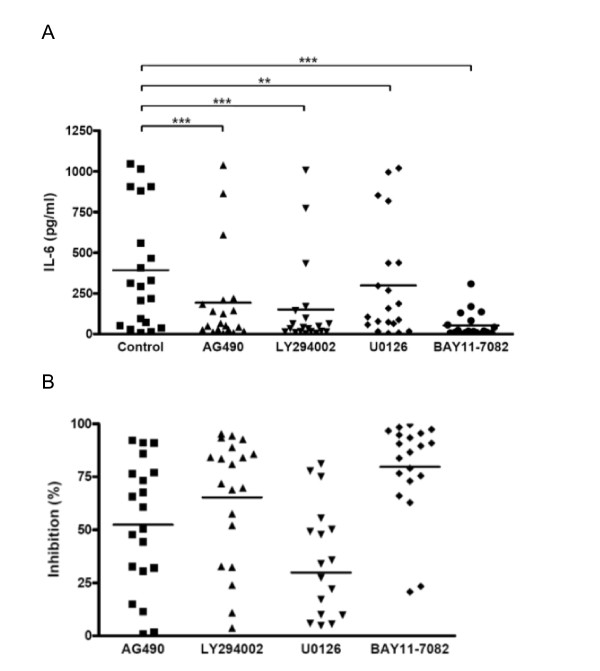
**Jak2/Stat3 pathway contributed to IL-6 autocrine production in clinically isolated lung cancer cells**. (A and B) Pharmacological inhibition of Jak2/Stat3, PI3-K/Akt, MEK/Erk and NF-κB pathways decreased the secretion of IL-6 in clinically isolated lung cancer cells. (A) Lung cancer cells were collected from MPE of 20 patients with lung adenocarcinomas. After resetting at 37°C for 1 hour, the cells were treated with AG490 (40 μM), LY294002 (20 μM), U0126 (5 μM), BAY11-7082 (20 μM) or medium alone for 24 hours. The culture supernatants were collected and IL-6 secretion was measured by ELISA. Each spot indicates the results of one sample. The graphs represent one experiment performed in triplicate represented as mean only. Paired t tests: **p < 0.01 and ***p < 0.001. (B) The inhibition rates of the four pharmacological inhibitors represent percent of inhibition, which was calculated by comparing IL-6 secretion in each tumor sample, with or without inhibitor treatment. It was defined as: (IL-6 level without inhibitor treatment - IL-6 level with inhibitor treatment) divided by IL-6 level without inhibitor treatment × 100%. Data represents the mean that each spot indicates the result of one sample done in triplicate.

Because previous studies suggesting Stat3 on IL-6 were all *in vitro *cell line studies, not clinical studies, we also wanted to find out whether IL-6 could be regulated by different combinations of pathways including Jak2/Stat3 in not just cell lines but also in the human body. We had previously found IL-6 levels to be increased in MPE of patients with lung cancer [2]. To do this, we pharmacologically inhibited the four IL-6 downstream pathways in 20 clinical samples of human lung cancer obtained from MPE. ELISA revealed that IL-6 was expressed in the conditioned medium of all samples, ranging from 16.58 ± 0.21 to 1016.47 ± 12.45 pg/ml (Figure 8A), with a mean of 393.14 pg/ml. The four aforementioned inhibitors significantly decreased IL-6 secretion in the clinically isolated cancer cells differently (U0126, p < 0.01; AG490, LY294002 and BAY11-7082, all p < 0.001) (Figure 8A). We further analyzed the percent of inhibition by each inhibitor on IL-6 secretion. BAY11-7082 had the greatest inhibitory activity on the autocrine production of IL-6 in the clinical samples (BAY11-7082 > LY294002 > AG490 > U0126) (Figure 8B).

## Discussion

IL-6 has been found to induce its own self-synthesis in many types of cells through transcriptional mechanisms [14,15]. Through this self-synthesis, the secreted IL-6 may induce further IL-6 production in cancer cells in which IL-6 is commonly produced. The IL-6 downstream signaling pathways MEK/Erk, PI3-K/Akt and NF-κB have been also found to be important regulators of IL-6 expression [12,16,18]. Several studies have noted an association between the most well-known IL-6 downstream pathway Jak2/Stat3 and expression of IL-6 as well [20,21], but direct proof has been lacking. Some studies, not specifically designed to study this relationship, have found some indication that there may be such a relationship, though some have not. Stat3 decoy oligonucleotide inhibited the expression of IL-6 and IL-10 mRNA [40] and Stat3 siRNA decreased the expression of IL-6, IL-10 and VEGF in melanoma cells [25], while the introduction of Stat3 siRNA did not inhibit Cox-2-induced IL-6 expression in the lung cancer cell A549 [41] and inhibition of Stat3 using antisense oligonucleotide and dominant-negative form of Stat3 in mouse cancer cells increased the expression of IL-6 [29]. Thus, we designed a series of biochemical and genetic studies of various established cancer cell lines and clinically isolated cancer cells to directly investigate the regulatory role of Stat3 on IL-6.

We found that blocking Jak2/Stat3 pathway as well as blocking the well-known PI3-K/Akt, MEK/Erk, and NF-κB pathways decreased IL-6 autocrine production in AS2 cells. We found that there was a clear association between Stat3 activation status and IL-6 expression pattern as well as paclitaxel resistance in AS2-derived cells and that knocked-down Stat3 by siRNA or shRNA decreased IL-6 expression in AS2 cells. Moreover, we also found that Stat3 also contributed to the elevation of IL-6 in drug resistant cell lines (KB-CPT100 and MCF-7/ADR) and that Jak2/Stat3 pathway cooperated with other IL-6 downstream pathways to regulate the expression of IL-6 in various drug resistant cancer cell lines and in clinically isolated lung cancer cells. Therefore, we clearly proved that Jak2/Stat3 together with the well characterized IL-6 downstream MEK/Erk, PI3-K/Akt and NF-κB pathways, jointly and differentially, regulates the autocrine production of IL-6 in a broad spectrum of established cancer cell lines as well as in clinical lung cancer samples.

Jak2/Stat3, as well as PI3-K/Akt, MEK/Erk, and NF-κB, are key signal pathways involved in cell survival. The blockage of these pathways by inhibitors or siRNAs may reduce cell survival. Thus, the reduction on IL-6 production by inhibitors or siRNAs might be indirectly caused by a reduced cell survival. We therefore investigated the effect of inhibitors and siRNAs on cell survival at the same treatment doses and periods as that used in the ELISA assay in all the tested cell lines. The siRNA transfection did not affect cell viability in any of the tested cells (Figure 4D and Additional file 3, Figure S3A-S3C) and the majority of inhibitors had only limited suppressive effect on cell viability (below 25%) (Additional file 1, Figure S1A-S1H) except that the PI3-K/Akt pathway inhibitor LY294002 had more suppressive activity on the cellular viability by 30 to 50% (Additional file 1, Figure S1C-S1F). However, LY294002 induced much greater (75 to 90%) decrease of IL-6 in these cells (Figure 7A to 7D). There is only one exception that the AG490-induced reductions of cell survival and IL-6 secretion were both about 30% in KB-7D cells (Additional file 1, Figure S1F and Figure 7D). Therefore, the reduction of cell survival might have major contribution to the suppression of IL-6 secretion by AG490 in this cell line. Taken together, we concluded that the reduction of IL-6 by pharmacological inhibitors and siRNAs used in this study are mainly caused by their specific effects on the targets rather than from the suppression of cellular viability.

In addition to Jak2/Stat3 pathway, PI3-K/Akt and MEK/Erk could also contribute to the regulation of IL-6 autocrine production in cancer cells. Thus, the three major down-stream pathways of IL-6 might crosstalk in the regulation of IL-6 autocrine production in cancer cells. In the experiment to test this possibility, we found that these three major IL-6 down-stream pathways were activated by the stimulation of IL-6 with different activating kinetics. There is no significant relationship between each other was found (Additional file 4, Figure S4A and S4B). Though the three pharmacological inhibitors could effectively inhibit both the basal and IL-6 induced phosphorylation of their targeting signal pathway, respectively, in AS2 cells (Additional file 4, Figure S4C and S4D), there was no off-target inhibition effect except that AG490 slightly increased the phosphorylation of Erk. This result is consistent with previous observation of the other studies [42]. However, AG490 still effectively decreased IL-6 expression in AS2 cells in spite of the slight increase of Erk phosphorylation. Therefore, Jak2/Stat3, MEK/Erk and PI3-K/Akt pathways individually contribute to the regulation of IL-6 autocrine production in cancer cells.

In the pharmacological experiments, Akt and Erk inhibitors significantly decreased IL-6 production in various cancer cells. To confirm these findings, we used siRNA against Akt1, Erk1 and Erk2 in AS2 cells. All of these siRNAs could effectively knock-down the expression of their targets (Additional file 5, Figure S5A and S5B) without affecting cell survival (Additional file 3, Figure S3D and S3E). Knocking-down Akt1 significantly decreased IL-6 secretion in AS2 cells (Additional file 5, Figure S5C). Knocking-down Erk1 significantly decreased IL-6 secretion but knocking-down Erk2 increased IL-6 secretion. The combinational knocking-down of Erk1 and Erk2 resulted in a limited reduction of IL-6 secretion only, compared to the mock and scramble siRNA control groups (Additional file 5, Figure S5D). We observed events of compensation that knocking-down of Erk1 induces an increase of phosphorylation on Erk2 and knocking-down of Erk2 induces an increase of phosphorylation on Erk1 (Additional file 5, Figure S5B). The limited reduction of IL-6 secretion by the combinational treatment may be caused by the compensation effect. Similarly, Lefloch *et al*. had also reported the compensational induction of Erk phosphorylation caused by siRNA knocking-down [43], which supports our speculation. Because, in our study, the siRNA approach is not an idea method to suppress Erk phosphorylation, we used another MEK/Erk inhibitor PD98059 to exclude the possible non-specific activity from U0126. The PD98059 effectively inhibited the phosphorylation of Erk1 and Erk2 (Additional file 5, Figure S5E) and decreased IL-6 secretion dose-dependently in AS2 cells (Additional file 5, Figure S5F). The treatment did not compromise cell survival (Additional file 1, Figure S1I). Collectively, these results confirm that both PI3-K/Akt and MEK/Erk pathways contribute to the regulation of IL-6 autocrine production in cancer cells.

Most studies investigating the regulation of IL-6 expression were performed in cell lines or animal models. In the present study, we took cancer cells from MPE of lung cancer patients and found that IL-6 regulation in human lung cancer samples to be similar to that in cancer cell lines. We found that the NF-κB pathway was the most important, but not an essential, regulator of IL-6 secretion in the tested cancer samples and that Jak2/Stat3 pathway contributed substantially to the regulation of IL-6 secretion in many cancer samples. Different cancer cells utilize different combinations of signals to orchestrate IL-6 autocrine production (Figure 8A and 8B). None of the tested signal pathways was found to be responsible for the regulation of IL-6 autocrine production alone. Instead, the IL-6 downstream signal pathways, including Jak2/Stat3, co-cooperated to control the IL-6 autocrine production in the cancer cells we tested.

In the literature, Stat3 siRNA did not affect COX-2-induced IL-6 expression in A549 cells [41]. In our study, however, knocking-down Stat3 with Stat3 siRNAs resulted in a decrease in IL-6 expression in AS2 cells and two drug resistant cancer cell lines (KB-CPT100 and MCF-7/ADR). To further evaluate this difference in findings, we also studied the effect of Stat3 on IL-6 expression in A549 cells. We found that Stat3 siRNA (Stat3#1) effectively knocked-down the expression of total amount of Stat3 protein and Stat3 phosphorylation (Additional file 6, Figure S6A) without affecting cell survival (Additional file 3, Figure S3F) but it did not decrease the secretion of IL-6 in A549 (Additional file 6, Figure S6B). Consistently, our biochemical studies, which showed limited side effects on cell survival (Additional file 1, Figure S1J), also demonstrated that inhibition of Jak2/Stat3 pathway did not reduce the secretion of IL-6 in A549 cells, but inhibition of NF-κB and PI3-K/Akt pathways did (Additional file 6, Figure S6C). Our knock-down studies of AS2, MCF-7/ADR, and KC-CPT100 cells and our pharmacological inhibition experiments with seven established cell lines and 20 clinical samples revealed that Stat3 did in fact affect expression of IL-6 in most of the cancer cells we tested.

In Stat3-null mouse embryonic fibroblasts, S3F up-regulated IL-6 mRNA expression suggesting that unphosphorylated Stat3 plays a role in regulating IL-6 expression [44]. In our study, however, treatment with A490 or over-expression of S3F inhibited Stat3 phosphorylation (Figure 2B and 3A) and reduced IL-6 expression (Figure 2A and 2C, and Figure 3B and 3E) in the Stat3 active AS2 cells. Similarly, AG490 treatment also decreased the IL-6 secretion in various drug resistant cancer cells exhibiting constitutively active Stat3 (Figure 7A to 7E). We hypothesized that unphosphorylated Stat3 may have a basal activity in the regulation of IL-6 expression but tyrosine phosphorylated Stat3 has better activity in the induction of IL-6 expression.

To date, no Stat3 binding site has yet been identified in IL-6 promoter. Using prediction software, we were also unable to find any specific Stat3 binding site 5 kb upstream from the transcriptional start site of IL-6 promoter. However, in the promoter experiments, we showed that a transient transfection of S3C plasmide into AS2 cells increased IL-6 promoter luciferase activity. On the contrary, the transient transfection of S3F plasmid or treatment with AG490 reduced IL-6 promoter luciferase activity in AS2 cells (Figure 2D and 3C). These results suggest that Stat3 might regulate IL-6 transcription at the promoter level. Stat3 has been reported to induce the expression of AP-1 proteins (JunB and c-Fos) [45,46] and C/EBPα, β and δ [47-49]. The AP-1 and C/EBP transcriptional factors are major regulators of IL-6 expression [13]. Therefore, Stat3 may increase the expression of IL-6 indirectly through the regulation of these transcriptional factors. However, it may do so directly by interacting with other transcription factors and co-localizing to IL-6 promoter at non-consensus sites. For example, Stat3 has been shown to interact directly with NF-κB forming a complex that synergistically promotes target genes expression [50,51]. Stat3 could also cooperate with C/EBPs [47], CREB [52], or AP-1 [53,54] to regulate target gene expression by binding to either its consensus sites or the non-consensus regions [51,55]. Regardless of how Stat3 contributes to the regulation of IL-6 expression, Stat3 DNA-binding activity is required. Our study demonstrates that over-expression of S3D suppresses IL-6 expression in AS2 cells (Figures 3B and 3D). That S3D is unable to bind to DNA suggests that Stat3 DNA binding activity plays an important role in the regulation of IL-6 expression. Our results will, however, need to be confirmed by further studies that further seek to uncover underlying mechanisms.

Consistent with previous literature [30,39], we found that drug resistant cancer cells secreted more IL-6 secretion than the parental cells (Figures 6 and 7), and not only NF-κB, PI3-K/Akt and MEK/Erk but also Jak2/Stat3 pathway contributed to the autocrine production of IL-6 in these cells. In the AS2-derived cells with different Stat3 activation statuses, we found a clear association among Stat3 activation status, IL-6 autocrine production and paclitaxel resistance. Similarly, the AS2 cells stably expressing Stat3 shRNA expressed less IL-6 mRNA, secreted less IL-6 protein, and were more sensitive to paclitaxel than the parental and vector-control cells (Figures 5B, 5C, and 5D). Paclitaxel resistance in these two cells could be modestly restored by adding exogenous IL-6 (Figure 5D), indicating that the IL-6-induced paclitaxel resistance is mediated by both Stat3-dependent and Stat3-independent pathways. By targeting Stat3, we may directly inhibit Stat3-dependent drug resistant mechanisms and inhibit Stat3-independent drug resistant mechanisms indirectly by decreasing IL-6 autocrine production in cancer cells simultaneously.

## Conclusions

In a series of biochemical and genetic studies, we clearly showed that Jak2/Stat3 pathway, together with other well characterized IL-6 downstream signal pathways, regulates the autocrine production of IL-6 in lung cancer cells and various drug resistant cancer cells. We also provided the first evidence that Stat3 participates in the regulation of IL-6 autorcine production in clinical samples. Collectively, our data show that Stat3 is one of the pivotal factors contributing to the regulation of autocrine production of IL-6 in cancer cells. Because the IL-6 feed-forward loop plays important role in the pathogenesis of inflammation-induced cancer as well as the drug resistance of cancer cells, the regulation of Stat3 could potentially be used to suppress IL-6 autocrine production in cancer cells.

## Abbreviations used

Erk: extracellular signal-related kinase; IL-6: interleukin-6; Jak: Janus kinase; MPE: malignant pleural effusion; PI3-K: phosphatidylinositol 3-kinase; Stat: signal transducer and activator of transcription

## Competing interests

The authors declare that they have no competing interests.

## Authors' contributions

WLH performed most experiments and wrote initial draft of the paper. HHY established AS2-derived Stat3 mutant cells. CCL and WWL participated in the collection of MPE from lung cancer patients. JYC contributed to the establishment and the assay of the drug resistant cancer cells. WTC contributed to the siRNA and shRNA associated experiments. WCS designed experiments and wrote final version of paper. All authors read and approved the final manuscript.

## Authors' information

WLH, *Ph.D. (Post-doctoral research fellow, molecular biologist)*

HHY, *Ph.D. (Post-doctoral research fellow, molecular biologist)*

CCL, *M.D. (Visiting staff member, pulmonologist)*

WWL, *M.D. (Associate-principle investigator, thoracic surgeon)*

JYC, *M.D. (Principle investigator, medical oncologist)*

WTC, *Ph.D. (Associate-principle investigator, molecular biologist*)

WCS, *M.D. (Principle investigator, medical oncologist)*

## Supplementary Material

Additional file 1 **Figure S1: The effect of pharmacological inhibitors treatment on cell survival in all the tested cells**. (A) Pharmacological inhibition of Jak2/Stat3, PI3-K/Akt, MEK/Erk and NF-κB pathways did not affect the viability of AS2 cells. AS2 cells were seeded for 24 hours and then treated with or without the Jak2/Stat3 inhibitor (AG490, 40 μM), the PI3-K/Akt inhibitor (LY294002, 20 μM), the MEK/Erk inhibitor (U0126, 5 μM), or the NF-κB inhibitor (BAY11-7082, 20 μM) for 24 hours. The cell viability was analyzed by MTT assay. (B) Increasing doses of AG490 showed limited effect on cell survival of AS2 cells. AS2 cells were seeded for 24 hours and then treated with the indicated doses of AG490 for 24 hours. The cell viability was analyzed by MTT assay. (C to H) The effect of pharmacological inhibition of Jak2/Stat3, PI3-K/Akt, MEK/Erk and NF-κB pathways on cell survival in drug resistant cancer cells. All cells were seeded for 24 hours and then treated with AG490 (40 μM), LY294002 (20 μM), U0126 (5 μM), BAY11-7082 (20 μM), or medium alone for 24 hours. The cell viability was analyzed by MTT assay. (I) Increasing doses of PD98059 showed limited effect on cell survival of AS2 cells. AS2 cells were seeded for 24 hours and then treated with the indicated doses of PD98059 for 24 hours. The cell viability was analyzed by MTT assay. (J) The effect of pharmacological inhibition of Jak2/Stat3, PI3-K/Akt, MEK/Erk and NF-κB pathways on cell survival in A549 cells. A549 cells were treated with AG490 (40 μM), LY294002 (20 μM), U0126 (5 μM), BAY11-7082 (20 μM) or medium alone for 24 hours. The cell viability was analyzed by MTT assay. The graphs (A-J) show the results as mean ± SEM. Click here for file

Additional file 2 **Figure S2: Knocking-down Stat3 by transient transfection with the second synthesized siRNA also decreased IL-6 expression**. (A) Transient transfection with the second Stat3 siRNA (Stat3#2) also effectively knocked-down Stat3. AS2 cells were left untreated as controls (C), transfected with nothing as mocks (M), or transfected with two different doses of scramble control siRNA or Stat3 siRNA (Stat3#2). The cells were incubated for 72 hours and then cell lysates were collected. The total amount of Stat3 protein and Stat3 phosphorylation level (pStat3-Y705) were analyzed by Western blot analysis. (B) Transient transfection with Stat3 siRNA decreased IL-6 secretion. 72 hours after transfection, the medium was replaced and culture supernatants were collected 3, 8 and 24 hours afterwards. IL-6 secretion was measured by ELISA. The graph represents the results as mean ± SEM. Student's t tests, *p < 0.05; **p < 0.01. For a clearer demonstration, statistical significances are shown for the 24-hour time points only. Click here for file

Additional file 3 **Figure S3: The effect of siRNA transfection on cell survival in all the tested cells**. (A) Transient transfection with the second Stat3 siRNA (Stat3#2) did not affect cell survival in AS2 cells. AS2 cells were left untreated as controls (C), transfected with nothing as mocks (M), or transfected with two different doses of scramble control siRNA or Stat3 siRNA (Stat3#2). The cells were incubated for 72 hours and then the cell viability was analyzed by MTT assay. (B and C) Transient transfection with Stat3 siRNA did not affect cell survival in KB-CPT100 and MCF-7/ADR cells. Cells were left untreated as controls (C), transfected with nothing as mocks (M), or transfected with 50 nM of scramble control siRNA or Stat3 siRNA (Stat3#1). The cells were incubated for 72 hours and then the cell viability was analyzed by MTT assay. (D and E) Transient transfection with the Akt1, Erk1, or Erk2 siRNA did not affect cell survival in AS2 cells. AS2 cells were transfected with nothing as mocks (M), or transfected with scramble control siRNA or Akt1siRNA, or Erk1siRNA, or Erk2 siRNA or co-transfected with Erk1siRNA and Erk2 siRNA (Erk1 + Erk2 siRNA). The cells were incubated for 72 hours and then the cell viability was analyzed by MTT assay. (F) Transient transfection with Stat3 siRNA did not affect cell survival in A549 cells. A549 cells were left untreated as controls (C), transfected with nothing as mocks (M), or transfected with 50 nM of scramble control siRNA or Stat3 siRNA (Stat3#1). The cells were incubated for 72 hours and then the cell viability was analyzed by MTT assay. The graphs (A-F) show the results as mean ± SEM. Click here for file

Additional file 4 **Figure S4: The three major IL-6 down-stream pathways could be activated by the stimulation of IL-6 with different activating kinetics that no significant relationship was found**. (A and B) The three major IL-6 down-stream pathways could be activated by the stimulation of IL-6 with different activating kinetics in both AS2 and KB-CPT100 cells. Cells were seeded for 24 hours and then treated with IL-6 (20 ng/ml). Cell lysates were collected at indicated time points and the activation of Stat3, Akt or Erk was evaluated by Western blot analysis. (C) AG490 effectively inhibited both the basal and IL-6 induced Stat3 activation with limited off-target effect. AS2 cells were seeded for 24 hours and then pre-treated with or without AG490 (40 μM) for 12 h following by treatment with or without IL-6 (20 ng/ml) for 15 min. Cell lysates were collected and the activation of Stat3, Akt or Erk was evaluated by Western blot analysis. (D) LY294002 and U0126 effectively inhibited both the basal and IL-6 induced Akt and Erk activation without off-target effect respectively. AS2 cells were seeded for 24 hours and then pre-treated with or without LY294002 (10 μM) or U0126 (5 μM) for 1 h following by treatment with or without IL-6 (20 ng/ml) for 15 min. Cell lysates were collected and the activation of Stat3, Akt or Erk was evaluated by Western blot analysis. Click here for file

Additional file 5 **Figure S5: Knocking-down Akt and Erk by transient transfection with synthetic siRNAs altered IL-6 secretion**. (A) Transient transfection with the Akt1 siRNA effectively knocked-down Akt. AS2 cells were transfected with nothing as mocks (M), or transfected with scramble control siRNA or Akt1 siRNA. The cells were incubated for 72 hours and then cell lysates were collected. The total amount of Akt1, Akt protein and Akt phosphorylation level (pAkt-S473) were analyzed by Western blot analysis. (B) Transient transfection with the Erk1 and Erk2 siRNA effectively knocked-down Erk. AS2 cells were transfected with nothing as mocks (M), or transfected with scramble control siRNA, or Erk1 siRNA, or Erk2 siRNA or co-transfected with Erk1 siRNA and Erk2 siRNA (Erk1 + Erk2 siRNA). The cells were incubated for 72 hours and then cell lysates were collected. The total amount of Erk protein and Erk phosphorylation level (pErk) were analyzed by Western blot analysis. (C) Transient transfection with Akt1 siRNA decreased IL-6 secretion. 72 hours after transfection, the medium was replaced and culture supernatants were collected 24 hours afterwards. IL-6 secretion was measured by ELISA. (D) Transient transfection with Erk1 and Erk2 siRNA altered IL-6 secretion. 72 hours after transfection, the medium was replaced and culture supernatants were collected 24 hours afterwards. IL-6 secretion was measured by ELISA. (E) PD98059 inhibited Erk phosphorylation in a dose-dependent manner. AS2 cells were seeded for 24 hours and then treated with the indicated doses of PD98059 for 1 hour. Its effect on Erk phosphorylation was analyzed by Western blot analysis. (F) PD98059 inhibited IL-6 secretion in a dose-dependent manner. AS2 cells were seeded for 24 hours and then treated with the indicated doses of PD98059 for 24 hours. Its effect on IL-6 secretion was analyzed by ELISA. The graphs (C, D and F) show the results as mean ± SEM. Student's t tests, **p < 0.01, and ***p < 0.001. Click here for file

Additional file 6 **Figure S6: Stat3 did not participate in the regulation of IL-6 in A549 cells**. (A) Transient transfection with the Stat3 siRNA (Stat3#1) also effectively knocked-down Stat3 in A549 cells. A549 cells were left untreated as controls (C), transfected with nothing as mocks (M), or transfected with 50 nM of scramble control siRNA or Stat3 siRNA (Stat3#1). The cells were incubated for 72 hours and then cell lysates were collected. The total amount of Stat3 protein and Stat3 phosphorylation level (pStat3-Y705) were analyzed by Western blot analysis. (B) Stat3 did not participate in IL-6 regulation in A549 cells. Medium replacement was performed 72 hours after transfection. Culture supernatants were collected 3, 8, or 24 hours after medium replacement. IL-6 secretion was measured by ELISA. The graph represents the results as mean ± SEM. (C) NF-κB and PI3-K/Akt, but not Jak2/Stat3 pathway regulated IL-6 secretion in A549 cells. A549 cells were treated with AG490 (40 μM), LY294002 (20 μM), U0126 (5 μM), BAY11-7082 (20 μM) or medium alone for 1, 3, 8, or 24 hours. The culture supernatants were collected at the indicated time points. IL-6 secretion was determined by ELISA. The graph represents the results as mean ± SEM. Student's t tests, ***p < 0.001. For a clearer demonstration, the statistical significance was only shown at the 24-hour time point. Click here for file
