# Supplementary figures and images for: Signal transducer and activator of transcription 3 activation up-regulates interleukin-6 autocrine production: a biochemical and genetic study of established cancer cell lines and clinical isolated human cancer cells
Source: Mol Cancer. 2010 Dec 2;9:309. doi: 10.1186/1476-4598-9-309 (PMC3027602; doi:10.1186/1476-4598-9-309)

## Slide 1
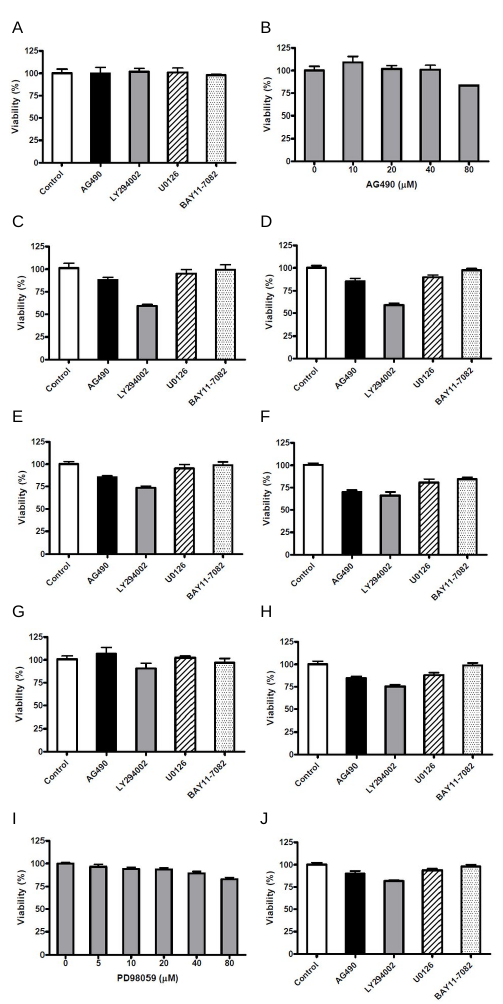

A
B
C
D
E
F
G
H
I
J

Supplement: Additional file 1 — Figure S1: The effect of pharmacological inhibitors treatment on cell survival in all the tested cells. (A) Pharmacological inhibition of Jak2/Stat3, PI3-K/Akt, MEK/Erk and NF-κB pathways did not affect the viability of AS2 cells. AS2 cells were seeded for 24 hours and then treated with or without the Jak2/Stat3 inhibitor (AG490, 40 μM), the PI3-K/Akt inhibitor (LY294002, 20 μM), the MEK/Erk inhibitor (U0126, 5 μM), or the NF-κB inhibitor (BAY11-7082, 20 μM) for 24 hours. The cell viability was analyzed by MTT assay. (B) Increasing doses of AG490 showed limited effect on cell survival of AS2 cells. AS2 cells were seeded for 24 hours and then treated with the indicated doses of AG490 for 24 hours. The cell viability was analyzed by MTT assay. (C to H) The effect of pharmacological inhibition of Jak2/Stat3, PI3-K/Akt, MEK/Erk and NF-κB pathways on cell survival in drug resistant cancer cells. All cells were seeded for 24 hours and then treated with AG490 (40 μM), LY294002 (20 μM), U0126 (5 μM), BAY11-7082 (20 μM), or medium alone for 24 hours. The cell viability was analyzed by MTT assay. (I) Increasing doses of PD98059 showed limited effect on cell survival of AS2 cells. AS2 cells were seeded for 24 hours and then treated with the indicated doses of PD98059 for 24 hours. The cell viability was analyzed by MTT assay. (J) The effect of pharmacological inhibition of Jak2/Stat3, PI3-K/Akt, MEK/Erk and NF-κB pathways on cell survival in A549 cells. A549 cells were treated with AG490 (40 μM), LY294002 (20 μM), U0126 (5 μM), BAY11-7082 (20 μM) or medium alone for 24 hours. The cell viability was analyzed by MTT assay. The graphs (A-J) show the results as mean ± SEM. [file 1476-4598-9-309-S1.PPT]

## Slide 1
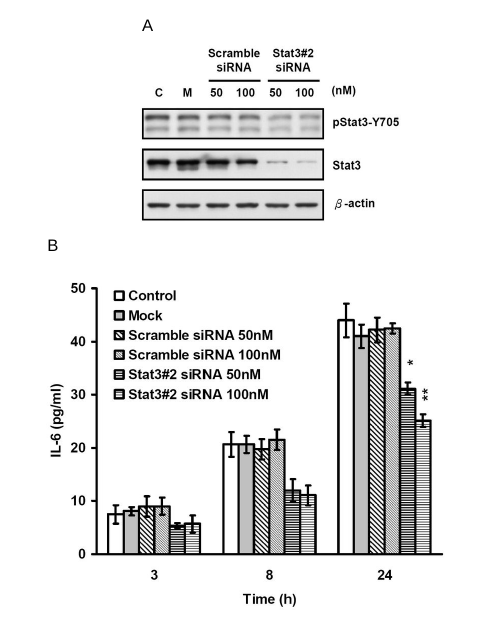

A
B

Supplement: Additional file 2 — Figure S2: Knocking-down Stat3 by transient transfection with the second synthesized siRNA also decreased IL-6 expression. (A) Transient transfection with the second Stat3 siRNA (Stat3#2) also effectively knocked-down Stat3. AS2 cells were left untreated as controls (C), transfected with nothing as mocks (M), or transfected with two different doses of scramble control siRNA or Stat3 siRNA (Stat3#2). The cells were incubated for 72 hours and then cell lysates were collected. The total amount of Stat3 protein and Stat3 phosphorylation level (pStat3-Y705) were analyzed by Western blot analysis. (B) Transient transfection with Stat3 siRNA decreased IL-6 secretion. 72 hours after transfection, the medium was replaced and culture supernatants were collected 3, 8 and 24 hours afterwards. IL-6 secretion was measured by ELISA. The graph represents the results as mean ± SEM. Student's t tests, *p < 0.05; **p < 0.01. For a clearer demonstration, statistical significances are shown for the 24-hour time points only. [file 1476-4598-9-309-S2.PPT]

## Slide 1
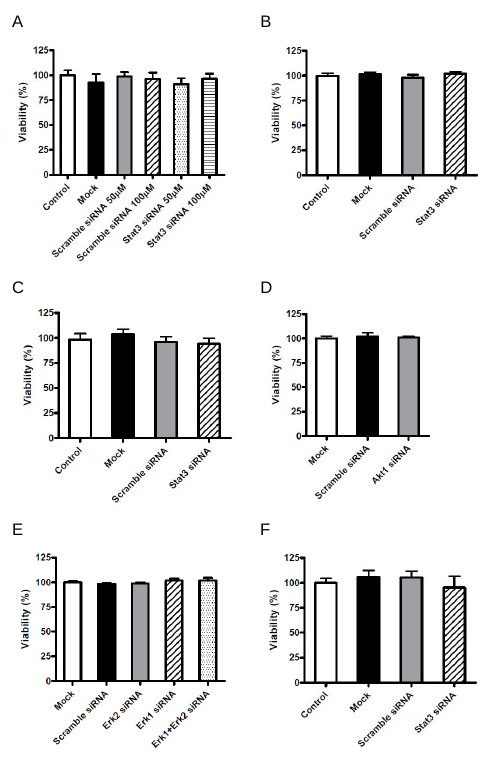

A
B
C
D
E
F

Supplement: Additional file 3 — Figure S3: The effect of siRNA transfection on cell survival in all the tested cells. (A) Transient transfection with the second Stat3 siRNA (Stat3#2) did not affect cell survival in AS2 cells. AS2 cells were left untreated as controls (C), transfected with nothing as mocks (M), or transfected with two different doses of scramble control siRNA or Stat3 siRNA (Stat3#2). The cells were incubated for 72 hours and then the cell viability was analyzed by MTT assay. (B and C) Transient transfection with Stat3 siRNA did not affect cell survival in KB-CPT100 and MCF-7/ADR cells. Cells were left untreated as controls (C), transfected with nothing as mocks (M), or transfected with 50 nM of scramble control siRNA or Stat3 siRNA (Stat3#1). The cells were incubated for 72 hours and then the cell viability was analyzed by MTT assay. (D and E) Transient transfection with the Akt1, Erk1, or Erk2 siRNA did not affect cell survival in AS2 cells. AS2 cells were transfected with nothing as mocks (M), or transfected with scramble control siRNA or Akt1siRNA, or Erk1siRNA, or Erk2 siRNA or co-transfected with Erk1siRNA and Erk2 siRNA (Erk1 + Erk2 siRNA). The cells were incubated for 72 hours and then the cell viability was analyzed by MTT assay. (F) Transient transfection with Stat3 siRNA did not affect cell survival in A549 cells. A549 cells were left untreated as controls (C), transfected with nothing as mocks (M), or transfected with 50 nM of scramble control siRNA or Stat3 siRNA (Stat3#1). The cells were incubated for 72 hours and then the cell viability was analyzed by MTT assay. The graphs (A-F) show the results as mean ± SEM. [file 1476-4598-9-309-S3.PPT]

## Slide 1
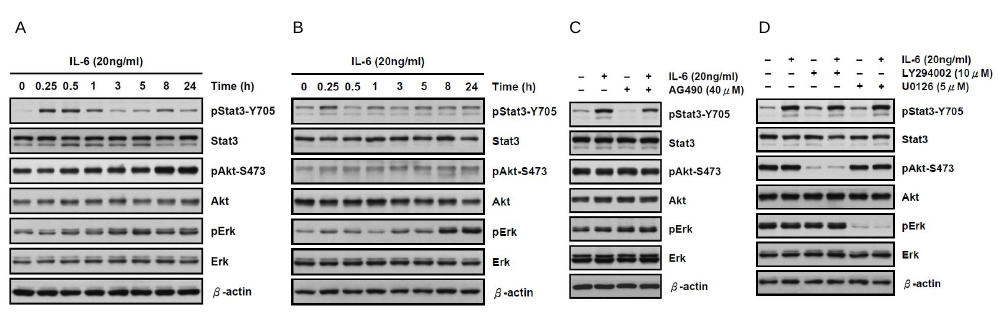

A
B
C
D

Supplement: Additional file 4 — Figure S4: The three major IL-6 down-stream pathways could be activated by the stimulation of IL-6 with different activating kinetics that no significant relationship was found. (A and B) The three major IL-6 down-stream pathways could be activated by the stimulation of IL-6 with different activating kinetics in both AS2 and KB-CPT100 cells. Cells were seeded for 24 hours and then treated with IL-6 (20 ng/ml). Cell lysates were collected at indicated time points and the activation of Stat3, Akt or Erk was evaluated by Western blot analysis. (C) AG490 effectively inhibited both the basal and IL-6 induced Stat3 activation with limited off-target effect. AS2 cells were seeded for 24 hours and then pre-treated with or without AG490 (40 μM) for 12 h following by treatment with or without IL-6 (20 ng/ml) for 15 min. Cell lysates were collected and the activation of Stat3, Akt or Erk was evaluated by Western blot analysis. (D) LY294002 and U0126 effectively inhibited both the basal and IL-6 induced Akt and Erk activation without off-target effect respectively. AS2 cells were seeded for 24 hours and then pre-treated with or without LY294002 (10 μM) or U0126 (5 μM) for 1 h following by treatment with or without IL-6 (20 ng/ml) for 15 min. Cell lysates were collected and the activation of Stat3, Akt or Erk was evaluated by Western blot analysis. [file 1476-4598-9-309-S4.PPT]

## Slide 1
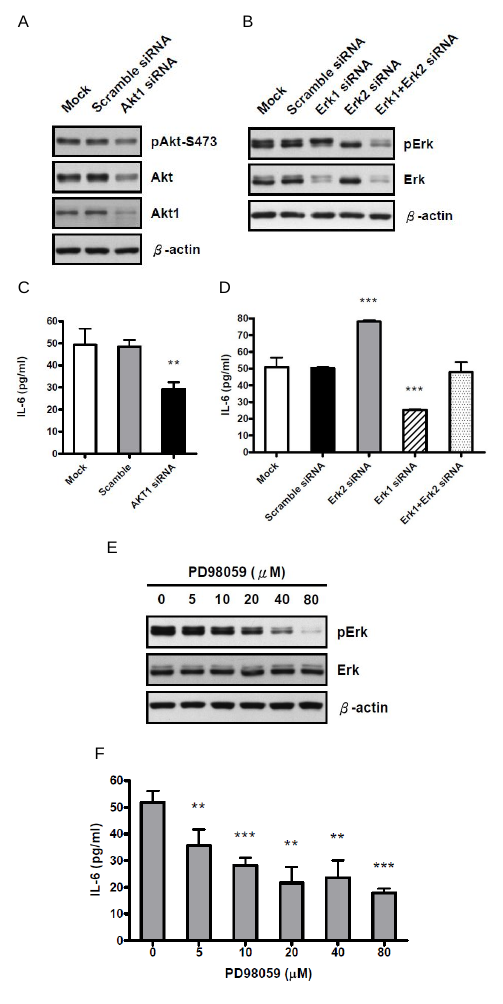

A
B
C
D
E
F

Supplement: Additional file 5 — Figure S5: Knocking-down Akt and Erk by transient transfection with synthetic siRNAs altered IL-6 secretion. (A) Transient transfection with the Akt1 siRNA effectively knocked-down Akt. AS2 cells were transfected with nothing as mocks (M), or transfected with scramble control siRNA or Akt1 siRNA. The cells were incubated for 72 hours and then cell lysates were collected. The total amount of Akt1, Akt protein and Akt phosphorylation level (pAkt-S473) were analyzed by Western blot analysis. (B) Transient transfection with the Erk1 and Erk2 siRNA effectively knocked-down Erk. AS2 cells were transfected with nothing as mocks (M), or transfected with scramble control siRNA, or Erk1 siRNA, or Erk2 siRNA or co-transfected with Erk1 siRNA and Erk2 siRNA (Erk1 + Erk2 siRNA). The cells were incubated for 72 hours and then cell lysates were collected. The total amount of Erk protein and Erk phosphorylation level (pErk) were analyzed by Western blot analysis. (C) Transient transfection with Akt1 siRNA decreased IL-6 secretion. 72 hours after transfection, the medium was replaced and culture supernatants were collected 24 hours afterwards. IL-6 secretion was measured by ELISA. (D) Transient transfection with Erk1 and Erk2 siRNA altered IL-6 secretion. 72 hours after transfection, the medium was replaced and culture supernatants were collected 24 hours afterwards. IL-6 secretion was measured by ELISA. (E) PD98059 inhibited Erk phosphorylation in a dose-dependent manner. AS2 cells were seeded for 24 hours and then treated with the indicated doses of PD98059 for 1 hour. Its effect on Erk phosphorylation was analyzed by Western blot analysis. (F) PD98059 inhibited IL-6 secretion in a dose-dependent manner. AS2 cells were seeded for 24 hours and then treated with the indicated doses of PD98059 for 24 hours. Its effect on IL-6 secretion was analyzed by ELISA. The graphs (C, D and F) show the results as mean ± SEM. Student's t tests, **p < 0.01, and ***p < 0.001. [file 1476-4598-9-309-S5.PPT]

## Slide 1
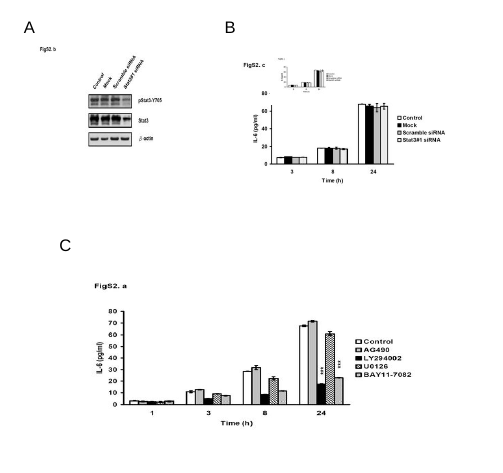

A
B
C

Supplement: Additional file 6 — Figure S6: Stat3 did not participate in the regulation of IL-6 in A549 cells. (A) Transient transfection with the Stat3 siRNA (Stat3#1) also effectively knocked-down Stat3 in A549 cells. A549 cells were left untreated as controls (C), transfected with nothing as mocks (M), or transfected with 50 nM of scramble control siRNA or Stat3 siRNA (Stat3#1). The cells were incubated for 72 hours and then cell lysates were collected. The total amount of Stat3 protein and Stat3 phosphorylation level (pStat3-Y705) were analyzed by Western blot analysis. (B) Stat3 did not participate in IL-6 regulation in A549 cells. Medium replacement was performed 72 hours after transfection. Culture supernatants were collected 3, 8, or 24 hours after medium replacement. IL-6 secretion was measured by ELISA. The graph represents the results as mean ± SEM. (C) NF-κB and PI3-K/Akt, but not Jak2/Stat3 pathway regulated IL-6 secretion in A549 cells. A549 cells were treated with AG490 (40 μM), LY294002 (20 μM), U0126 (5 μM), BAY11-7082 (20 μM) or medium alone for 1, 3, 8, or 24 hours. The culture supernatants were collected at the indicated time points. IL-6 secretion was determined by ELISA. The graph represents the results as mean ± SEM. Student's t tests, ***p < 0.001. For a clearer demonstration, the statistical significance was only shown at the 24-hour time point. [file 1476-4598-9-309-S6.PPT]
